# Biomineralization through a Symmetry-Controlled Oligomeric Peptide

**DOI:** 10.3390/biomimetics8080606

**Published:** 2023-12-14

**Authors:** Tatsuya Sakaguchi, Natsumi Nakagawa, Kenta Mine, Jose Isagani B. Janairo, Rui Kamada, James G. Omichinski, Kazuyasu Sakaguchi

**Affiliations:** 1 Laboratory of Biological Chemistry, Department of Chemistry, Faculty of Science, Hokkaido University, Sapporo 060-0810, Japan; sakaguchi_tatsuya@kurume-u.ac.jp (T.S.); n-nakagawa@sci.hokudai.ac.jp (N.N.); kenta.mine@outlook.com (K.M.); kamadar@sci.hokudai.ac.jp (R.K.); 2 Department of Chemistry, Kurume University School of Medicine, Kurume 830-0011, Japan; 3 Biology Department, De La Salle University, 2401 Taft Avenue, Manila 0922, Philippines; jose.isagani.janairo@dlsu.edu.ph; 4 Département de Biochimie et Médicine Moléculaire, Université de Montréal, Montréal, QC H3C 3J7, Canada

**Keywords:** biomineralization peptide, silver nanostructure, coiled-coil peptides, DNA-based scaffold, hexagonal disk

## Abstract

Biomineralization peptides are versatile tools for generating nanostructures since they can make specific interactions with various inorganic metals, which can lead to the formation of intricate nanostructures. Previously, we examined the influence that multivalency has on inorganic structures formed by p53 tetramer-based biomineralization peptides and noted a connection between the geometry of the peptide and its ability to regulate nanostructure formation. To investigate the role of multivalency in nanostructure formation by biomineralization peptides more thoroughly, silver biomineralization peptides were engineered by linking them to additional self-assembling molecules based on coiled-coil peptides and multistranded DNA oligomers. Under mild reducing conditions at room temperature, these engineered biomineralization peptides self-assembled and formed silver nanostructures. The trimeric forms of the biomineralization peptides were the most efficient in forming a hexagonal disk nanostructure, with both the coiled-coil peptide and DNA-based multimeric forms. Together, the results suggest that the spatial arrangement of biomineralization peptides plays a more important role in regulating nanostructure formation than their valency.

## 1. Introduction

The ability to control the shape and size of inorganic nanostructures is critical for material science and nanotechnology, since these properties have substantial impacts on the functional attributes of the synthesized materials [1,2,3,4]. It has been shown that material morphology influences a wide array of the functional properties of nanostructures, including optical, electrical, catalytic, and biological, among others [5,6,7,8,9,10,11]. The use of biomolecules, such as biomineralization peptides, for the synthesis of inorganic nanostructures has gained popularity due to their inherent capacity to self-assemble, combined with their ability to chelate a wide variety of different metal ions [12,13]. It has been shown that biomineralization peptides can be used to create a wide range of inorganic nanomaterials with remarkably diverse structures and chemical properties [14,15,16]. For example, biomineralization peptides have been used as capping agents, where they bind to the surface of the growing nanostructure [17]. Peptides and other biomolecules, such as DNA, can also assume the role of templates during nanostructure synthesis, wherein the biomolecular assembly impacts the morphology of the resulting nanostructure [18,19,20,21,22,23]. In an effort to further improve peptide-mediated biomineralization as a method for creating novel nanostructures, several key factors that govern biomineralization have been identified and used to help tune the properties of the resulting nanostructures. Some of these factors that have been incorporated into biomineralization peptides include the sequence, addition of post-translational modifications, control of the stoichiometry, and even the contents of the buffering solution used to prepare the nanostructures [24,25,26,27,28,29]. There are unique advantages to using peptide-based biomineralization and this helps distinguish it from more conventional synthetic methodologies. These advantages include its proficiency in self-assembly, the potential to easily incorporate a diverse array of modifications via their functional groups, and its capacity to efficiently facilitate mineral formation under benign conditions. Such attributes render it as a promising strategy for fabricating complex functional materials.

Previously, we have demonstrated that the spatial orientation and valency of a biomineralization peptide can have a huge impact on the resulting morphology and functional properties of the resulting nanostructure. In order to determine how a spatially fixed orientation and controlled valency of a peptide can regulate biomineralization, we examined the influence of these parameters on peptide binding affinity. In our previous study, we conjugated the silver biomineralization peptide minTBP-1 [30] to the tetramerization domain of the tumor suppressor p53 protein (p53Tet). Conjugation of minTBP-1 with the p53Tet resulted in the formation of a tetrahedral orientation for minTBP-1. As controls, monomeric and dimeric variants of the p53 peptide conjugated to minTBP-1 were also prepared and tested. The results demonstrated that the silver nanostructure produced using the tetrameric peptide formed a hexagonal plate, a morphology that did not reflect the peptide structure [27]. We further observed that the tetrameric minTBP-1 exhibited a binding preference for a silver {111} crystal surface, a property that was absent in both the monomeric and dimeric forms of the minTBP-1 peptides. These results highlighted the complexity of the synthesis process of nanostructure synthesis using oligomeric biomolecules and warranted further investigation into the roles that valency and spatial orientation play in the formation of nanostructures from biomineralization peptides. In this study, we have further examined the impact that peptide valency and spatial orientation have on biomineralization. Specifically, we employed coiled-coil peptides and DNA as alternative biomolecular scaffolds for forming biomineralization peptides in combination with the silver-binding minTBP-1 peptide. Based on the results with different combinations of valency and spatial arrangements, we have proposed a hypothesis that the geometrical arrangement of biomineralization peptides exerts a strong influence on nanoparticle growth. Our findings suggest that the spatial arrangement of the biomineralization peptide bound to the surface of the nanostructure surface outweighs valency in terms of regulating overall nanostructure growth.

## 2. Materials and Methods

### 2.1. Peptide Synthesis and Purification

The coiled-coil conjugated minTBP-1 peptides (TBP–CCs) were synthesized on an Applied Biosystems 433A automated peptide synthesizer using a standard Fmoc synthetic strategy on a rink amide resin. The peptide sequences are summarized in Table S1. The cleaved peptide obtained after treatment with reagent K (9 mL trifluoroacetic acid (TFA), 0.5 mL water, 0.5 mL phenol, 0.5 mL thioanisole, and 0.25 mL ethanedithiol) was purified to homogeneity using a Shimadzu LC-6AD HPLC equipped with 22 × 250 mm Vydac C8 column with a binary gradient of buffered CH_3_CN/H_2_O as the solvent system (Figure S1). The mass of the purified peptides were verified using an Applied Biosystems Voyager 4379 MALDI-TOF MS (Figure S2). Peptide concentrations were determined spectrophotometrically using the 280 nm extinction coefficient (ε_280_): for TBP–CC(Di) peptide, ε_280_ = 1490 M^−1^cm^−1^, corresponding to a single tyrosine; for TBP–CC(Tri) and TBP–CC(Tet) peptides, ε_280_ = 5500 M^−1^cm^−1^, corresponding to a single tryptophan.

### 2.2. Synthesis and Purification of TBP–DNA Fragment and Oligomer Formation

The minTBP-1 peptide (RKLPDA-GGC) was synthesized by the Fmoc solid-phase method using an automatic synthesizer (433A peptide synthesizer, Applied Biosystems, Waltham, MA, USA). A cysteine was incorporated and used to connect the peptide to the DNA oligonucleotide, and two glycines were used as a spacer between the peptide and DNA portions of the molecule. The cleaved minTBP-1 peptide (RKLPDA-GGC) was obtained after treatment with reagent K (9 mL trifluoroacetic acid (TFA), 0.5 mL water, 0.5 mL phenol, 0.5 mL thioanisole, and 0.25 mL ethanedithiol) and was purified using a Shimadzu LC-6AD HPLC equipped with a 22 × 250 mm Vydac C8 column with a binary gradient of buffered CH_3_CN/H_2_O as the solvent system.

Maleimide-oligonucleotides (Mal-DNA) were synthesized by mixing 3.1 mM aminated oligonucleotides and 31 mM NHS-Maleimide in HEPES buffer (pH 7.4) for 6 h at room temperature and purified by gel filtration chromatography (Sephadex G-10, volume 1.0 mL, column length 6.3 cm, Nipro syringe). The synthesized Mal-DNA was stored as a powder following lyophilization and dissolved in HEPES buffer when used for the next reaction. The concentration of the dissolved Mal-DNA was determined spectrophotometrically (UV-Vis) using an extinction coefficient (ε_280_) = 69800 M^−1^cm^−1^.

The purified minTBP-1 peptide and Mal-DNA were conjugated using a covalent cysteine–maleimide coupling strategy [16]. Briefly, 3.9 mM Mal-DNA and 78 mM minTBP-1 peptide were mixed in HEPES buffer (pH 7.4) for 6 h at room temperature. The synthesized TBP–DNA was purified to homogeneity using a Shimadzu PU-980 HPLC equipped with a 4 × 250 mm Vydac C8 column with a binary gradient of buffered CH_3_CN/H_2_O as the solvent system.

When the DNA-based minTBP-1 oligomers (TBP–DNAs) were formed, the amount of each DNA fragment was adjusted to coincide with the concentration of minTBP-1 to be added. The minTBP-1 concentration during the biomineralization reaction was 10 μM, and each single-stranded DNA was mixed at 5, 3.3, and 2.5 μM for the dimeric, trimeric, and tetrameric TBP–DNA, respectively. The prepared solution was heated in boiling water for 3 min and then allowed to cool until it reached room temperature. After the solution cooled to room temperature, the solution was stored at 4 °C.

### 2.3. Confirmation of TBP–DNA Structure Formation by Native-PAGE

Sample solutions were prepared by mixing the adaptor strand conjugated minTBP-1 (TBP-AS) and frame strands to achieve a concentration of 40 μM in 20 mM phosphate buffer (pH 7.4), yielding a final volume of 5 μL. A 20% polyacrylamide gel was prepared in-house by combining a 30% acrylamide/bis solution, 750 mM HEPES-NaOH buffer (pH 8.0), 10 mM EDTA-NaOH (pH 8.0), 10% ammonium persulfate (APS), and water in a ratio of 66.65:20:10:2.25:1:0.1, respectively. The gel was allowed to polymerize at 4 °C for approximately 1 h. Each sample was mixed with 6x loading dye and placed into the gel wells. The gel was run in 1× TBE buffer at a constant voltage of 150 V, maintaining a temperature of 4 °C until the dye front was approximately 1 cm from the bottom of the gel. Following electrophoresis, the gel was stained with ethidium bromide to visualize the DNA. Bands were observed and captured under UV light.

### 2.4. Thermal Stability Analysis by Circular Dichroism (CD) Spectroscopy

A Jasco-805 spectropolarimeter was employed for the CD measurements using a 1 mm path length quartz cell. CD spectra were recorded in phosphate buffer (20 mM sodium phosphate (pH 7.4)). For the thermal denaturation studies, spectra were recorded at discrete temperatures between 4 and 96 °C using a scan rate of 1 °C/min. The ellipticity was measured at 222 nm for TBP–CC samples (10 μM) and at 275 nm for TBP–DNA samples (20 μM) (Figures S3 and S4). The unfolding process of the oligomeric TBP–CCs and TBP–DNAs was modeled as a two-state transition, wherein the native oligomer directly converts to an unfolded monomer, consistent with previous descriptions [31,32]. The thermodynamic parameters of the oligomer were determined using the calculation functions as described by Mateu et al. [31].

### 2.5. Biomineralization Reaction

TBP–CC or TBP–DNA stock solutions were first added to a 20 mM HEPES-NaOH buffer (pH 7.4). Silver nitrate solution was introduced into the buffer to create a final concentration of 10 μM for TBP–CC and TBP–DNA, and 100 μM for silver ions. The biomineralization reaction was initiated by adding L-ascorbic acid to the reaction solution at a two-fold excess relative to the silver ions to generate the elemental silver. The solution was subsequently incubated at 20 °C for 48 h.

### 2.6. Electron Microscopy

The nanostructures resulting from the biomineralization reaction were isolated through centrifugation. The resuspended nanoparticles were then deposited onto a carbon-coated copper grid for structural analysis. The structural characterizations were performed using a Hitachi HD-2000 scanning transmission electron microscope (Hitachi, Tokyo, Japan) operated at an acceleration voltage of 200 kV. The surface morphology of nanostructures was examined under the scanning electron microscope (SEM) mode, whereas the finer details were observed using the transmission electron microscope (TEM) mode of the HD-2000 instrument.

## 3. Results

### 3.1. Design and Synthesis of the Oligomeric minTBP-1

#### 3.1.1. Coiled-Coil-Based Oligomer

**Figure 1:**
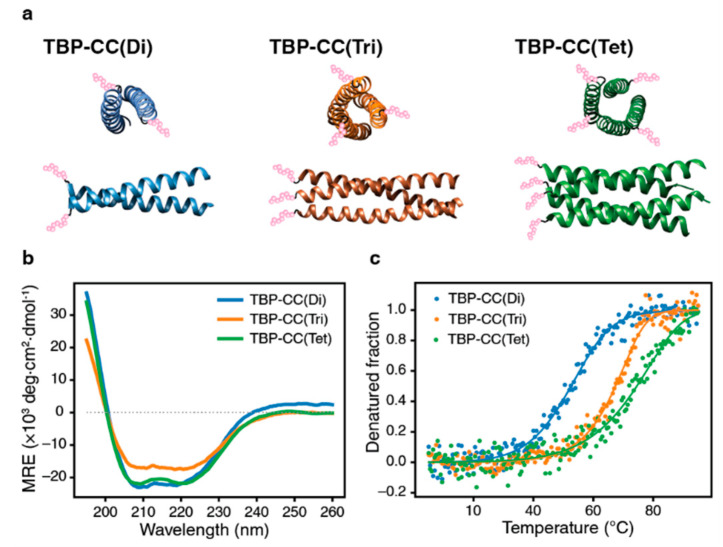
Validation of the specific oligomeric structure of the TBP–CCs peptides. (**a**) Illustrative schematic of the TBP–CC peptide structure, depicted by interconnected pink spheres representing the minTBP-1 peptide. (**b**) Circular Dichroism (CD) spectra of the three different TBP–CC peptides: dimeric form (blue), trimeric form (orange), and tetrameric form (green). Each spectrum displays a characteristic α-helical pattern, which is signified by double minima at 208 nm and 222 nm. (**c**) Thermal denaturation curves of the TBP–CC peptides: dimeric form (blue), trimeric form (orange), and tetrameric form (green). The fraction of denatured peptide for each form was calculated from the change in ellipticity at 222 nm as a function of temperature (Figure S3).

Coiled-coil peptides were used as the foundation for the preparation of the oligomeric biomineralization peptides due to their capacity to form a wide variety of different oligomeric configurations. The coiled-coil motif is a prevalent structural feature commonly found in natural proteins, and numerous natural as well as synthetic coiled-coil peptides have been characterized [33,34]. Our study focused on the design of fusion peptides composed of parallel dimeric [35], trimeric [36], and tetrameric [37] coiled-coil peptides. These peptides were fused to the C-terminus of minTBP-1, and the peptides were separated by a pair of glycine residues acting as a linker bridge between them (Table S1). TBP oligomers were assembled using parallel coiled-coil peptides as the scaffold, as this would facilitate the simultaneous interaction of all of the minTBP-1s of an oligomer with the silver surface. The spatial configuration of the minTBP-1 peptides was determined by forming coiled-coil-mediated oligomers. Specifically, in the dimeric TBP (TBP–CC(Di)) fusion peptide, the minTBP-1 peptide adopts a linear organization. Likewise, for the trimeric (TBP–CC(Tri)) and tetrameric (TBP–CC(Tet)) arrangements, the minTBP-1 peptides are structured in either a triangular or a square configuration, respectively (Figure 1a). The formation of oligomeric structures in TBP–CC peptides was verified by analyzing their circular dichroism (CD) spectra. The three different oligomeric TBP–CC peptide forms all displayed double minima at 208 nm and 222 nm, which is indicative of the α-helical structural arrangement, and this implies that they are adopting a coiled-coil structure (Figure 1b). The TBP–CC peptides were further analyzed to evaluate their relative thermostabilities. Thermodynamic parameters were deduced from CD-based denaturation curves, and the melting temperatures (*T*_m_) for TBP–CC(Di), TBP–CC(Tri), and TBP–CC(Tet) were determined to be 52.6 °C, 67.4 °C, and 74.5 °C, respectively. These data suggest that the peptides assemble into stable oligomers at 20 °C, a condition conducive for preparing silver nanoparticles. At this temperature, the calculated percentages of the oligomeric forms for TBP–CC(Di), TBP–CC(Tri), and TBP–CC(Tet) were determined to be 98.9%, 99.8%, and 99.7%, respectively (Figure 1c).

#### 3.1.2. DNA-Based Oligomer

**Figure 2:**
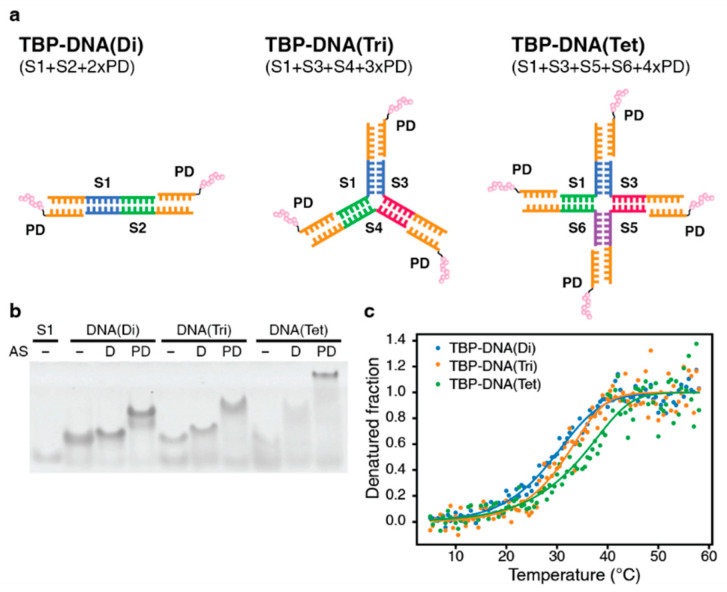
Validation of the specific oligomeric structure of the TBP–DNA products. (**a**) Schematic representation of the DNA-based oligomeric minTBP-1 structures (TBP–DNAs), where the minTBP-1 peptide is illustrated as a chain of interconnected pink spheres. (**b**) Native Polyacrylamide Gel Electrophoresis (Native-PAGE) of the TBP–DNA mixtures. The mixtures containing the DNA fragments designed to form a specific oligomer were separated electrophoretically using a native acrylamide gel. Each structure is represented in three forms: without an adaptor strand (-), with only an adaptor strand (D), and with TBP–DNA fragments (PD). (**c**) Thermal denaturation curves of the different TBP–DNA oligomeric forms: dimeric form (blue), trimeric form (orange), and tetrameric form (green). The fraction of denatured oligomer was calculated by measuring the changes in ellipticity at 275 nm relative to the change in temperature (Figure S4).

In addition to developing oligomeric peptides based on different coiled-coil scaffolds, we also attempted to engineer a series of different DNA-based oligomeric minTBP-1 peptides. To construct the peptide-DNA oligomers with varying oligomeric states (dimeric, trimeric, and tetrameric) that can direct the spatial arrangement of the attached biomineralization peptides, we initially fused minTBP-1 with a 6-mer DNA fragment (CGATAG) (Figure 2a). This conjugation was achieved by attaching the C-terminus of the minTBP-1 peptide to the DNA fragment using a covalent cysteine–maleimide bonding strategy to produce the chimeric peptide–DNA (PD) product. To produce the various oligomeric forms, we combined 18-mer DNA fragments (frame strand) with the PD fragment in specific combinations (Figure 2a, Table S2). Each frame strand consisted of three distinct 6-mer nucleotide segments. In each of the six fragment strands (S1–S6), the 3′ segment (CTATCG) of the oligonucleotide contained a sequence that matches the complementary sequence of the 6-mer DNA sequence present in the chimeric PD product. In addition, the 5′ and middle sections of the DNA oligonucleotides contained the sequence that is complementary to the sequences found in other frame strands. This complementary relationship enables the formation of dimeric, trimeric, and tetrameric complexes when PD is mixed with specific frame strands (Figure 2a). The oligomerization states of the TBP–DNA products were confirmed by Native-PAGE. The oligomers were formed by mixing the PD fragments with the correct combination of frame strands. The mixed solution of frame strands forming the dimer, trimer, and tetramer contained an upper band on the Native-PAGE gel when compared to the same length DNA fragment, and the additional shift was confirmed by the addition of the PD fragments (Figure 2b). Furthermore, the intensity levels of the bands increased as the number of oligomers increased. These results indicate that the frame strands adopted the intended oligomeric forms, and the oligomeric forms were further stabilized by the addition of the PD fragments. The relative stabilities of the DNA-based oligomers were also investigated by determining their thermostability based on CD measurements. The calculated melting temperature (*T*_m_) for the TBP–DNA(Di), TBP–DNA(Tri), and TBP–DNA(Tet) were 29.4 °C, 31.7 °C, and 34.8 °C, respectively, indicating that the DNA-based biomineralization peptides would be predominantly in their oligomeric forms under the conditions required for forming the silver nanoparticle (20 °C). The calculated percentages of the oligomeric form for TBP–DNA(Di), TBP–DNA(Tri), and TBP–DNA(Tet) were calculated to be 84.5%, 91.1%, and 90.2%, respectively (Figure 2c).

### 3.2. Silver Nanostructure Formation

#### 3.2.1. Coiled-Coil-Based Oligomer

**Figure 3:**
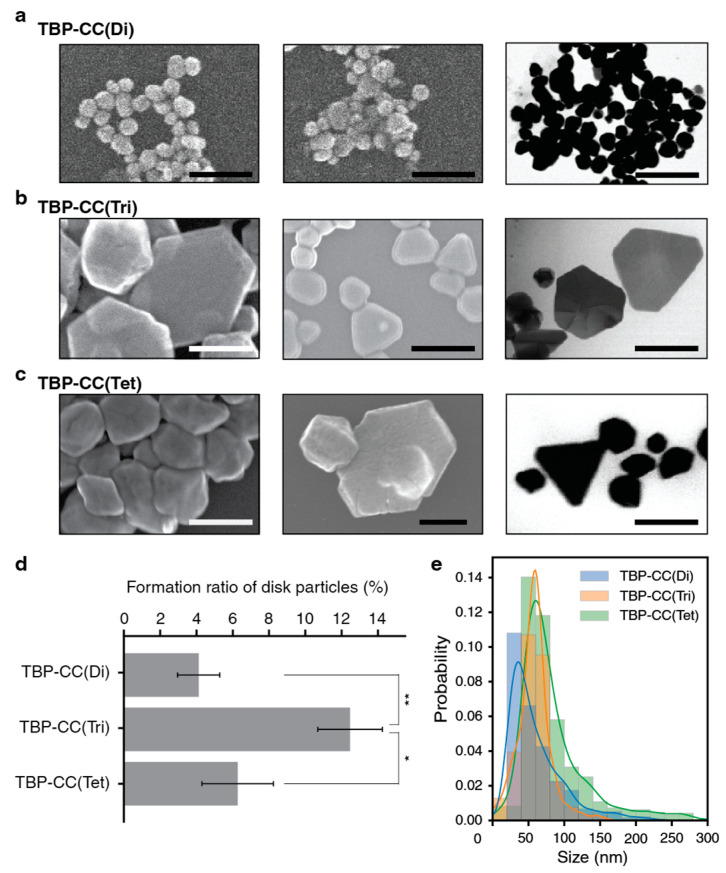
Silver nanostructures formed by the different oligomeric TBP–CC peptides. Representative Transmission Electron Microscopy (TEM)/Scanning Electron Microscopy (SEM) images of silver nanostructures formed by (**a**) TBP–CC(Di), (**b**) TBP–CC(Tri), and (**c**) TBP–CC(Tet), with a scale bar of 200 nm. (**d**) Proportions of silver nanoplate formed by TBP–CC(Di), TBP–CC(Tri), and TBP–CC(Tet) peptides. Error bars represent the standard error. Statistical significance between groups are as follows: * *p* < 0.1; ** *p* < 0.05. (**e**) Histogram and Kernel Density Estimate (KDE) plot of silver nanoparticle sizes produced by TBP–CC(Di) (blue), TBP–CC(Tri) (orange), and TBP–CC(Tet) (green). For panels (**d**,**e**), the number of particles analyzed for TBP–CC(Di), TBP–CC(Tri), and TBP–CC(Tet) was *n* = 542, *n* = 567, and *n* = 843, respectively.

To test the role of spatial organization on nanostructure formation, silver nanostructures were prepared using the TBP–CC peptides. The biomineralization reactions were performed in the presence of the mild reducing agent L-ascorbic acid and at a comparatively low temperature (20 °C). The TBP–CC peptides at a concentration of 10 μM were incubated with silver nitrate at a concentration of 100 μM for 48 h at 20 °C, after which the resulting nanostructures were examined using STEM (Figure 3a–c). In general, the spherical particles that were observed possessed rough surfaces, whereas the plate-like structures that formed had smoother surfaces. The silver nanoplates were observed following incubation of the three different oligomeric forms of the TBP–CC peptides, but more nanoparticles were observed with the trimeric form of the peptide in comparison to the more complex oligomeric form (Figure 3d). The formation ratio of silver nanoplates with the trimeric TBP–CC peptide stood at 13%, while the nanoplate formation ratios with dimeric and tetrameric TBP–CC peptides were 4% and 6%, respectively. Overall, there was a significant disparity between the trimeric form and the other two forms (*p* = 0.017, 0.080). The silver nanoparticles formed with the TBP–CC(Tri) peptide were the most uniform in terms of their size distribution, with the mean and median sizes being 58.0 nm and 58.2 nm, respectively. In contrast, the nanoparticles formed by the TBP–CC(Di) and TBP–CC(Tet) peptides exhibited a wider distribution in size and often possessed long tails. For the TBP–CC(Di) peptide, the mean and median particle sizes were 60.3 nm and 47.7 nm, whereas those formed in the presence of the TBP–CC(Tet) peptide were 84.0 nm and 67.9 nm (Figure 3e).

#### 3.2.2. DNA-Based Oligomer

**Figure 4:**
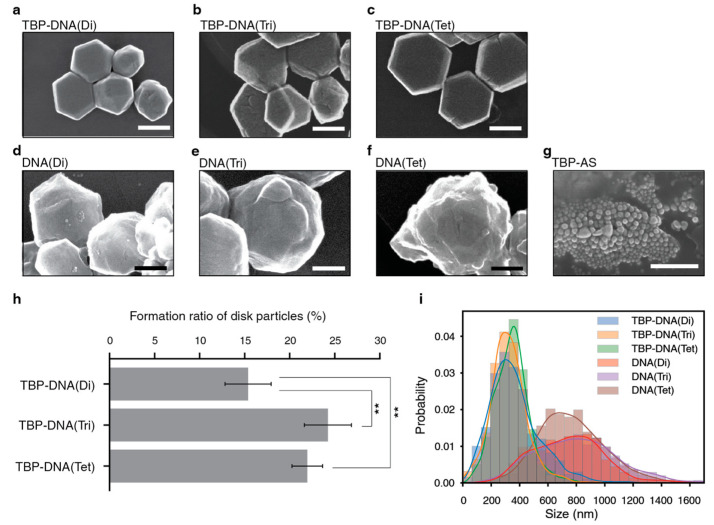
Silver nanostructures formed by different oligomeric forms of TBP–DNA. Representative Scanning Electron Microscopy (SEM) images of silver nanostructures formed by (**a**) TBP–DNA(Di); (**b**) TBP–DNA(Tri); (**c**) TBP–DNA(Tet); (**d**) DNA(Di); (**e**) DNA(Tri); (**f**) DNA(Tet); and (**g**) TBP-AS, with a scale bar of 200 nm. (**h**) The formation ratio for silver nanoplates facilitated by TBP–DNA(Di), TBP–DNA(Tri), and TBP–DNA(Tet). Error bars represent standard error. Statistical significance between groups is as follows: ** *p* < 0.05. (**i**) Histogram and Kernel Density Estimate (KDE) plot of the different sizes of silver nanoparticle produced by TBP–CC(Di) (blue), TBP–CC(Tri) (orange), TBP–CC(Tet) (green), DNA(Di) (red), DNA(Tri) (purple), and DNA(Tet) (brown). For panels (**h**,**i**), the number of particles analyzed for TBP–DNA(Di), TBP–DNA(Tri), TBP–DNA(Tet), DNA(Di), DNA(Tri), and DNA(Tet) was *n* = 1500, *n* = 1397, *n* = 1356, *n* = 973, *n* = 1071, and *n* = 1304, respectively. The histogram and KDE plot illustrating the silver nanoparticles produced by TBP-AS are presented in Figure S5.

To further examine the role of valency and spatial arrangement in nanostructure formation, silver nanostructures were fabricated using the different oligomeric forms of the TBP–DNA constructs under the same experimental conditions employed with the TBP–CC peptides. The dimeric, trimeric, and tetrameric forms of the TBP–DNA complexes all produced thin silver nanoplates of hexagonal or truncated triangular forms under the experimental conditions (Figure 4a–c). The ratios of silver nanoplate formation were very similar when comparing the trimeric (24%) and tetrameric (22%) forms of the TBP–DNA (Figure 4h), whereas the nanoplate formation ratio for the dimeric variant was significantly reduced at 15% (*p* = 0.036, 0.050). In contrast to what was observed with the different oligomeric forms of the TBP–CC peptides, the nanostructure sizes of the three different oligomeric forms of the TBP–DNA displayed almost identical size distributions.

Interestingly, the DNA-only complex, which intrinsically interacts with Ag^+^ [38,39], produced larger spherical particles with rough surfaces (Figure 4d–f,i), whereas minTBP-1 with adaptor strands (TBP-AS) and without frame strands created small spherical particles (Figure 4g and Figure S5). These observations indicate that the minTBP-1 peptides must be in an oligomeric structure to form hexagonal silver nanoplates. Additionally, the nanoplates observed in the TBP–DNA samples resembled closely those observed with the corresponding oligomeric forms of the TBP–CC peptides. This suggests that the increase in silver nanoplate formation by minTBP-1 results from the type of multimerization that is induced by the different scaffolds and not from the chemical composition of the specific scaffold molecules used.

## 4. Discussion

We have determined that the trimeric forms of TBP–CC peptides and TBP–DNAs are most effective for inducing the formation of silver nanoplates when attached to the minTBP-1 peptide. In previous works, we demonstrated that the minTBP-1 peptide conjugated to the p53 tetramerization domain peptide (p53Tet) also induced the formation of silver nanoplates [27]. Our previous study found that minTBP-1 exhibits a specific binding ability to silver {111} surfaces, suggesting that this binding specificity is essential for nanoplate formation. However, the relationship between the valence of multimerized minTBP-1 and the ability to form silver nanoplates was not clearly defined in this work because the steric hindrance caused by the tetrameric structure of TBP-p53Tet does not allow the four monomers of p53 attached to minTBP-1s to bind simultaneously in the same silver crystal plane during nanostructure growth.

In this study, we synthesized stable and rigid dimeric, trimeric, and tetrameric forms of the TBP peptide by conjugating it to different oligomeric forms of parallel coiled-coil peptides. In order to analyze the effect of different types of multimeric forms of TBP on silver biomineralization, we performed silver biomineralization using dimeric, trimeric, and tetrameric peptides. Interestingly, the trimeric form of the TBP peptide, TBP–CC(Tri), significantly increased the ratio of plate structure formation in silver particle formation compared to either the dimeric TBP–CC(Di) or the tetrameric TBP–CC(Tet). These results suggest that the structural arrangement of the TBP bound to the growing silver nanocrystal surface is an essential factor in regulating silver nanoplate growth rather than the valence of the TBP peptide. In general, triangular or hexagonal silver nanoplates were created by capping {111} crystal faces during their crystal growth [40,41]. It is worth noting that the TBPs are arranged in C3 symmetry in the trimeric form of the TBP–CC, whereas the arrangement of the silver atoms on the {111} surface is in C6 symmetry [42]. The C3 symmetry is contained within the C6 symmetry so that all TBP peptides of the trimeric TBP–CC(Tri) can bind to the silver surface using the same mode of binding. On the other hand, the TBPs are arranged in the C4 symmetry in the tetrameric form of the TBP–CC, which means there are at least two TBP–Ag binding modes with the tetrameric TPB-CC(Tet). The importance of the peptide conformation and the atomic arrangement of the inorganic surface for the peptide–inorganic surface interaction has been reported in molecular dynamics simulation studies [43,44,45] which supports our view that the control of the TBP peptide orientation by multimerization of the TBP–CC has a strong influence on the rate of formation of silver nanoplates. This consideration is supported by the fact that the TBP–CC is a crucial factor in the formation of silver nanoplates. In summary, it can be hypothesized that the ability to control nanocrystal growth is enhanced by matching the orientation of the capping agent to the atomic arrangement of its binding planes.

Furthermore, this study found that the formation of silver nanoplates was also enhanced when DNA was used as a scaffold to generate different oligomeric forms of minTBP-1. DNA is widely used to create nanomaterials and can produce a wide range of complex structures due to its structural freedom and ease of design as a scaffold. Examples of scaffolds formed with DNA include fixed gold nanoparticles in equivalent areal densities [16,21], dumbbell-like nanostructures [46], and nanoparticles arrayed along DNA strands [19]. The monomeric TBP–DNA (TBP-AS) and DNA alone were only able to create nanomaterials for distorted spherical silver nanoparticles, whereas silver nanoplate formation was significantly increased with the different multimeric forms of the TBP–DNA. Similar to what was observed using multimerization with the different coiled-coil peptides, the largest number of silver nanoplates were observed with the trimeric form of the TBP–DNA. These results suggest that the enhancement of silver nanoplate formation via oligomerization of minTBP-1 depends not on the chemical composition of the scaffold molecules but on the oligomerization state or the spatial arrangement.

In this study, we analyzed the effect of changing the oligomeric form (dimeric to tetrameric) of minTBP-1s on silver nanoplate formation using either a coiled-coil scaffold or a DNA scaffold. We found that the trimeric forms of both scaffoldings were the most efficient at forming silver nanoplates in combination with minTBP-1. The preference for the trimeric form can be explained for the most part by the relationship between the orientation of the multimerized capping agent and the atomic arrangement of the targeted bonding crystal planes. Although further validation is needed to support this theory, this study provides new factors to consider when designing novel capping agents for future nanocrystal fabrication. Furthermore, as the present study suggests that the effect of multimerization of minTBP-1 occurs independent of the chemical composition of the attached scaffolding molecule, it is strongly expected that the precise and exhaustive valence and orientation-controlling ability of biomolecules can be used to elucidate the control mechanism of nanocrystal formation by oligomerized biomineralizing peptides. Silver nanostructures, including nanoplates, are renowned for their diverse applications [47]. The distinct optical properties of silver nanoplates, like surface plasmon resonance [3,41], along with their physiological attributes, including antibacterial and anticancer activities [11,48], are intricately tied to their size and shape. Consequently, our research offers promising avenues for developing a range of functional silver nanostructures.

## Data Availability

All relevant data are within the paper and online supplemental data.

## Supplementary Materials

The following supporting information can be downloaded at: https://www.mdpi.com/article/10.3390/biomimetics8080606/s1. Table S1: Peptide sequences of TBP–CC peptides; Table S2: DNA sequences of frame strands; Figure S1: HPLC profiles of the synthesized peptides; Figure S2: MALDI-TOF MS spectra of the synthesized peptides; Figure S3: Change in ellipticity at 222 nm as a function of temperature for TBP–CC peptides; Figure S4: Change in ellipticity at 275 nm as a function of temperature for TBP–DNA oligomers; Figure S5: Histogram and Kernel Density Estimate (KDE) plot of silver nanoparticle sizes produced by TBP–DNAs.
