# Supplementary material for: Biomineralization through a Symmetry-Controlled Oligomeric Peptide
Source: Biomimetics (Basel). 2023 Dec 14;8(8):606. doi: 10.3390/biomimetics8080606 (PMC10742239; doi:10.3390/biomimetics8080606)
Supplement: Supplementary file 1 [file biomimetics-08-00606-s001.zip › biomimetics-2749953-supplementary.pdf]

Supplementary information for

Biomineralization through a symmetry-controlled oligomeric peptide

Tatsuya Sakaguchi [1,2,†], Natsumi Nakagawa [1,†], Kenta Mine [1], Jose Isagani B. Janairo [3], Rui Kamada [1], James G. Omichinski [4*], and Kazuyasu Sakaguchi [1,*]

1. Laboratory of Biological Chemistry, Department of Chemistry, Faculty of Science, Hokkaido University, Sapporo 060-0810, Japan; sakaguchi_tatsuya@kurume-u.ac.jp (T.S.); n-nakagawa@sci.hokudai.ac.jp (N.N.); kenta.mine@outlook.com (K.M.), kamadar@sci.hokudai.ac.jp (R.K.)
2. Department of Chemistry, Kurume University School of Medicine, Kurume 830-0011, Japan.
3. Biology Department, De La Salle University, 2401 Taft Avenue, 0922 Manila, Philippines.
4. Département de Biochimie et Médicine Moléculaire, Université de Montréal, H3C 3J7 QC, Canada; jg.omichinski@umontreal.ca

* Correspondence: jg.omichinski@umontreal.ca (J.G.O.); kazuyasu@sci.hokudai.ac.jp (K.S.)

† These authors contributed equally to this work.

Content:

- **Table S1:** Peptide sequences of TBP-CC peptides.
- **Table S2:** DNA sequences of frame strands.
- **Figure S1:** HPLC profiles of the synthesized peptides.
- **Figure S2:** MALDI-TOF MS spectra of the synthesized peptides.
- **Figure S3:** Change in ellipticity at 222 nm as a function of temperature for TBP-CC peptides
- **Figure S4:** Change in ellipticity at 275 nm as a function of temperature for TBP-DNA oligomers.
- **Figure S5:** Histogram and Kernel Density Estimate (KDE) plot of silver nanoparticle sizes produced by TBP-DNAs.

Table S1. Peptide sequences of TBP-CC peptides

Name	Sequence
TBP-CC(Di)	H-RKLPDAGGRMKQLEDKVEELLSKNYHLENEVARLKKLVGER-NH_2
TBP-CC(Tri)	H-RKLPDAGGSGIDQEQNNLTRLIEAQIHELQLTQWKIKQLLARIL-NH_2
TBP-CC(Tet)	H-RKLPDAGGGELAAIKQELAAIKKELAAIKWELAAIKQGAG-NH_2

Table S2. DNA sequences of frame strands used in TBP-DNA constructs

Strand ID	Sequence
S1	5′-AACCTG CGAAGT ctatcg-3′
S2	5′-ACTTCG CAGGTT ctatcg-3′
S3	5′-ACTTCG GATGCA ctatcg-3′
S4	5′-TGCATC CAGGTT ctatcg-3′
S5	5′-TGCATC GACCAT ctatcg-3′
S6	5′-ATGGTC CAGGTT ctatcg-3′

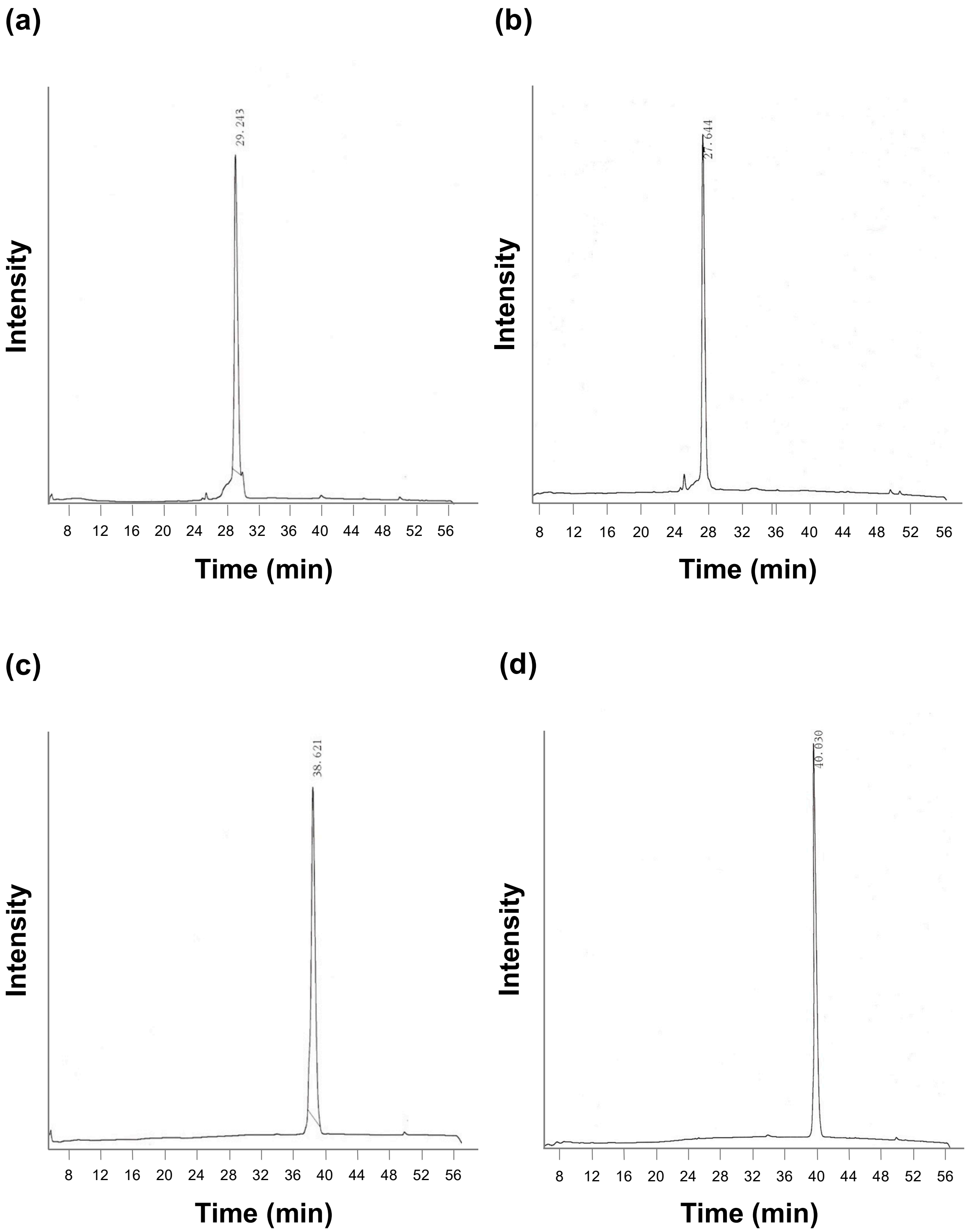
(a)
29.243
Intensity
8 12 16 20 24 28 32 36 40 44 48 52 56
Time (min)
(b)
27.644
Intensity
8 12 16 20 24 28 32 36 40 44 48 52 56
Time (min)
(c)
38.621
Intensity
8 12 16 20 24 28 32 36 40 44 48 52 56
Time (min)
(d)
40.030
Intensity
8 12 16 20 24 28 32 36 40 44 48 52 56
Time (min)

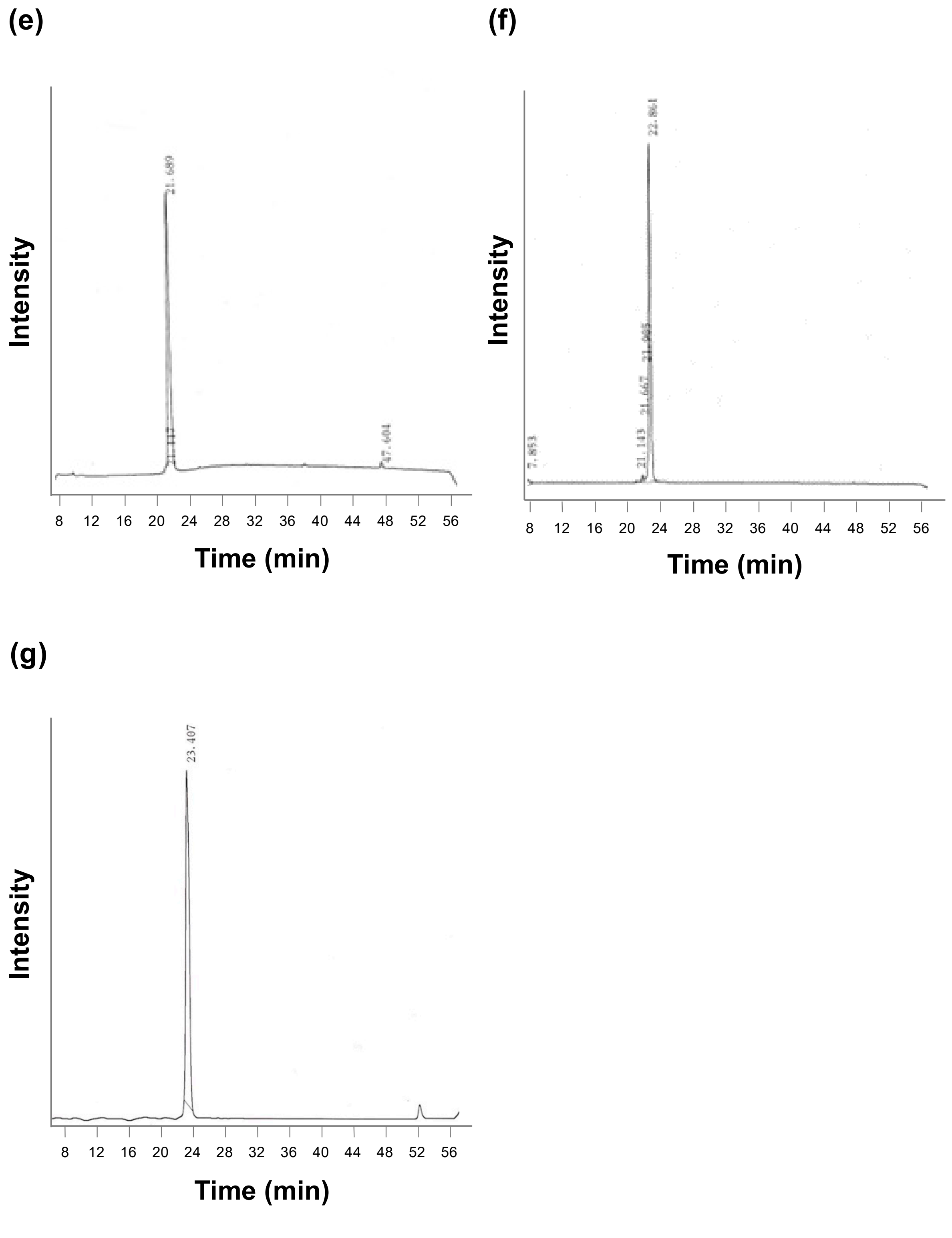

Figure S1. HPLS profiles of the synthesized peptides. (a) TBP-CC(Di), (b) CC(Di), (c) TBP-CC(Tri), (d) CC(Tri), HPLS profiles of the synthesized peptides. (e) TBP-CC(Tet), (f) CC(Tet), (g) TBP-GlyGlyCys

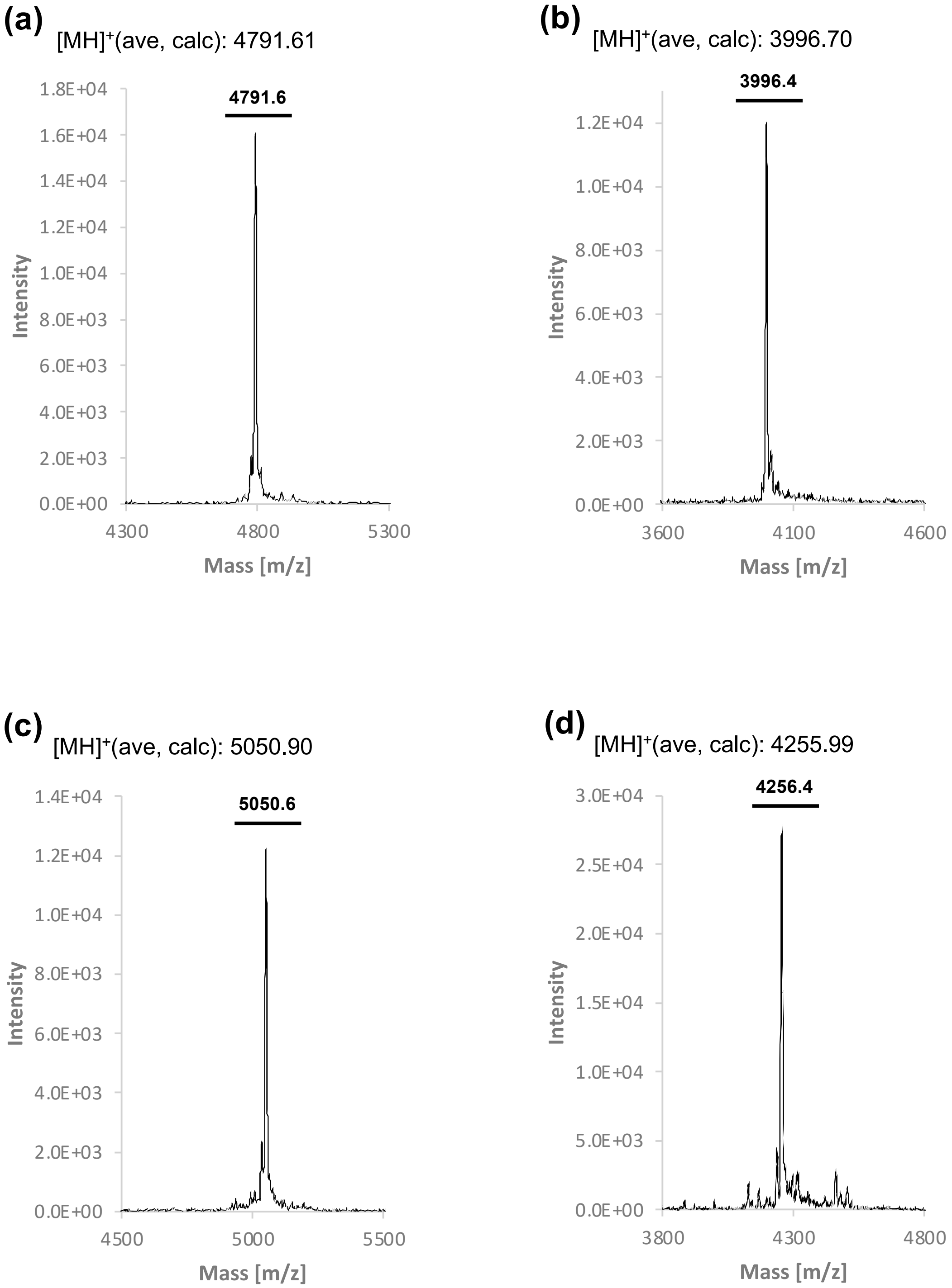
(a)
[MH]+(ave, calc): 4791.61
4791.6
Intensity
Mass [m/z]
1.8E+04
1.6E+04
1.4E+04
1.2E+04
1.0E+04
8.0E+03
6.0E+03
4.0E+03
2.0E+03
0.0E+00
4300
4800
5300
(b)
[MH]+(ave, calc): 3996.70
3996.4
Intensity
Mass [m/z]
1.2E+04
1.0E+04
8.0E+03
6.0E+03
4.0E+03
2.0E+03
0.0E+00
3600
4100
4600
(c)
[MH]+(ave, calc): 5050.90
5050.6
Intensity
Mass [m/z]
1.4E+04
1.2E+04
1.0E+04
8.0E+03
6.0E+03
4.0E+03
2.0E+03
0.0E+00
4500
5000
5500
(d)
[MH]+(ave, calc): 4255.99
4256.4
Intensity
Mass [m/z]
3.0E+04
2.5E+04
2.0E+04
1.5E+04
1.0E+04
5.0E+03
0.0E+00
3800
4300
4800

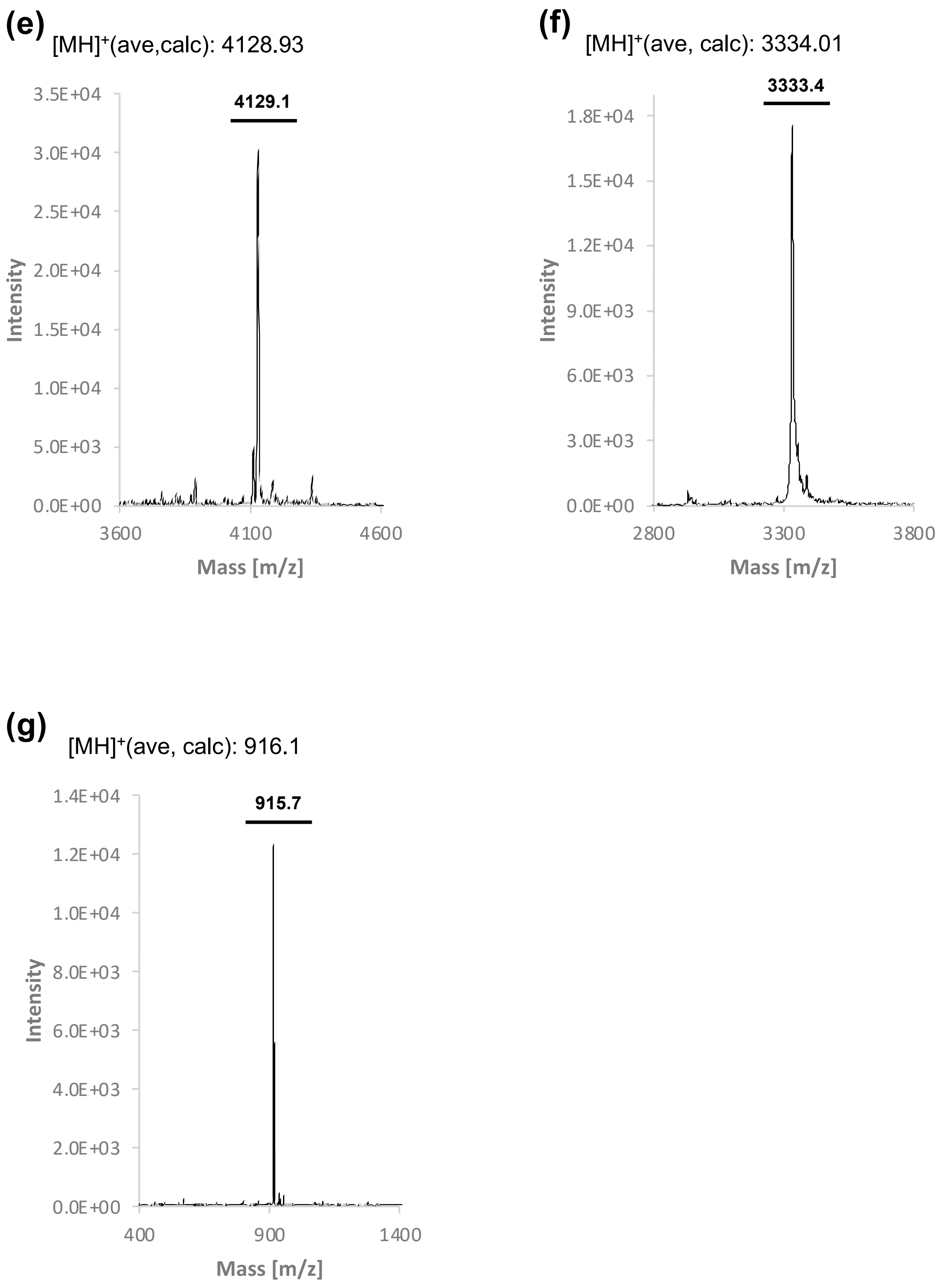

Figure S2. MALDI-TOF MS spectraof the synthesized peptides. (a) TBP-CC(Di), (b) CC(Di), (c) TBP-CC(Tri), (d) CC(Tri), MALDI-TOF MS spectra of the synthesized peptides. (e) TBP-CC(Tet), (f) CC(Tet), (g) TBP-GlyGlyCys

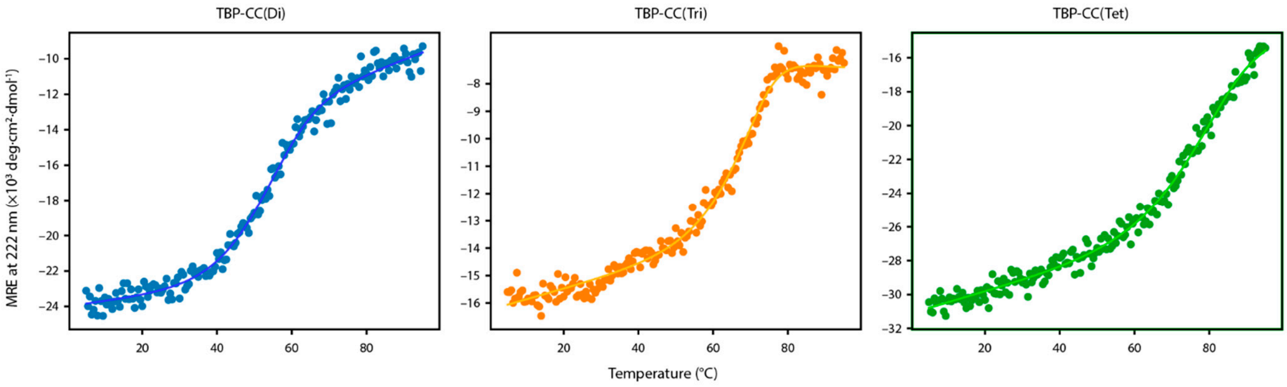

Figure S3. Change in ellipticity at 222 nm as a function of temperature for TBP-CC peptides. The temperature-dependent curves are shown for the TBP-CC(Di) peptide (**left panel**), the TBP-CC(Tri) peptide (**center panel**), and the TBP-CC(Tet) peptide (**right panel**). Peptide solutions were prepared at a concentration of 10 µM in a 20 mM phosphate buffer (pH 7.4). Temperature variation was monitored from 4ºC to 96ºC, with a scanning rate of 1ºC per minute.

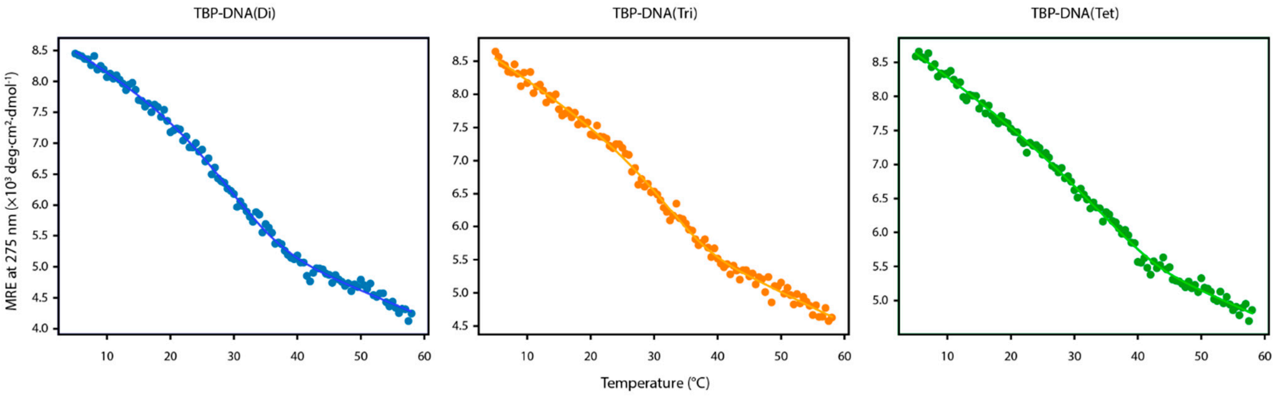

Figure S4. Change in ellipticity at 275 nm as a function of temperature for TBP-DNA oligomers. The temperature-dependent curves are shown for the TBP-DNA(Di) peptide (**left panel**), the TBP-DNA(Tri) peptide (**center panel**), and TBP-DNA(Tet) peptide (**right panel**). The solutions were prepared with a concentration of 20 µM monomer strands in a 20 mM phosphate buffer (pH 7.4). The temperature ranged from 4ºC to 58ºC, and the scan rate was set at 1ºC per minute.

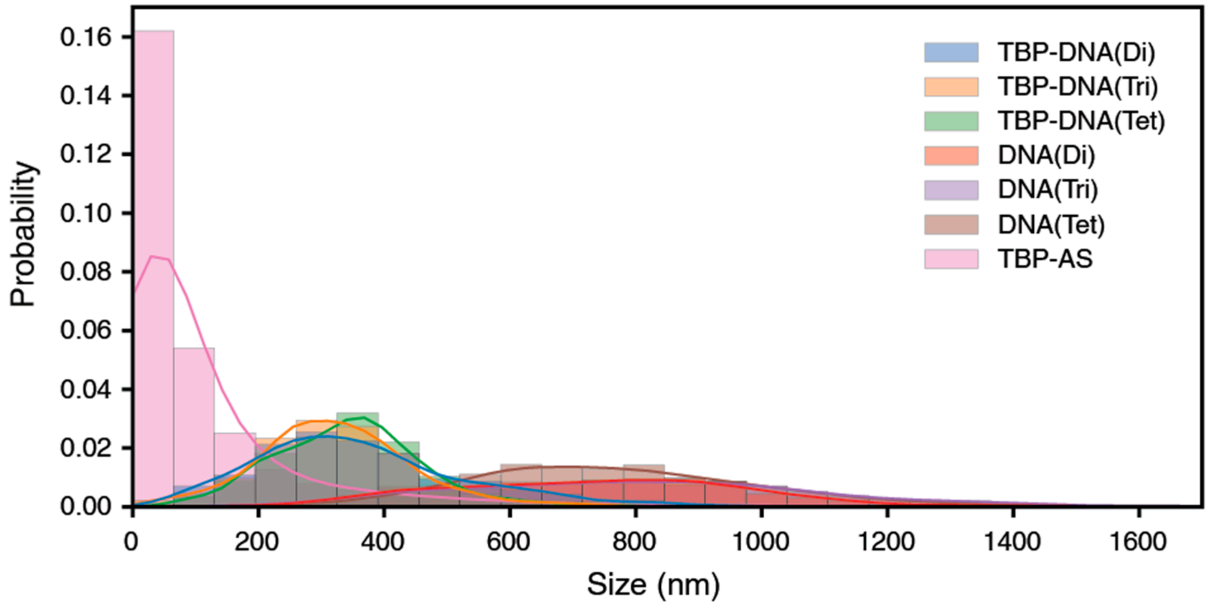

Figure S5. Histogram and Kernel Density Estimate (KDE) plot of silver nanoparticle sizes produced by TBP-DNA(Di) (blue), TBP-DNA(Tri) (orange), TBP-DNA(Tet) (green), DNA(Di) (red), DNA(Tri) (purple), DNA(Tet) (brown), and TBP-AS (pink). The number of particles analyzed for TBP-DNA(Di), TBP-DNA(Tri), TBP-DNA(Tet), DNA(Di), DNA(Tri), DNA(Tet), and TBP-SA were N=1500, N=1397, N=1356, N=973, N=1071, N=1304, and N=3035, respectively.
